# A comparative evaluation of green colloidal silver variants for enhanced stability and bioactivity

**DOI:** 10.1038/s41598-025-30332-7

**Published:** 2025-12-05

**Authors:** Federico Trotta, Danielle Winning, Sophie Sadiatoonasa, Seyedeh Fatemeh Mirpoor, Stella Lignou, Sameer Khalil Ghawi, Dimitris Charalampopoulos

**Affiliations:** 1 Metalchemy Limited, 71-75 Shelton Street, London, WC2H 9JQ UK; 2 Department of Food and Nutritional Sciences, University of Reading, P.O. Box 226, Whiteknights, Reading, RG6 6AP UK

**Keywords:** Colloidal silver, Sustainable technology, Eco-friendly synthesis, Scalable green materials, Alternative antimicrobials, Green chemistry, Sustainability, Nanoscience and technology, Nanoscale materials, Nanoparticles, Chemistry, Chemical engineering

## Abstract

Colloidal silver (CS) is at the forefront of enhancing novel consumer products formulations & packaging. However, conventional synthesis methods pose challenges, including toxicity, high costs, and poor colloidal stability. Green synthesis using plant extracts offers a sustainable alternative by leveraging natural bioactive compounds. This study compares CS synthesized via a patented plant-based method (CS_BX3_), Lemon Juice (CS_LJ_), and Green Tea (CS_GT_), with a commercial reference (CS_Ref_). Characterization using UV–Vis, DLS, SEM, Zeta Potential, and EDS assessed particle size, stability, and composition under stress conditions: long-term storage (60 days), varying temperatures (4–37 °C), pH (3–11), and phosphate-buffered saline (PBS). Antioxidant activity was evaluated and antimicrobial efficacy against *E.coli* and *Listeria* to determine MIC. CS_BX3_ and CS_LJ_ showed superior stability under various stress conditions CS_BX3_ retained colloidal stability both at low temperatures (3.97% size reduction at 4 °C) and in PBS (14.2% in SPR intensity). CS_LJ_ displayed strong steric stabilization, showing minimal size variation in PBS. Zeta potential and SEM analyses confirmed that CS_BX3_ maintained dispersion and particle uniformity across neutral and alkaline pH, while CS_GT_ showed significant aggregation**.** CS_GT_ and CS_BX3_ showed the most potent antimicrobial and antioxidant properties (20% higher antioxidant activity than other CS solutions and over 90% bacterial eradication within 24 h). These results highlight the potential of green-synthesized CS for food, biomedical, and personal care applications.

**Supplementary Information:**

The online version contains supplementary material available at 10.1038/s41598-025-30332-7.

## Introduction

Product preservation plays a vital role in reducing waste, ensuring safety, and extending the lifespan of consumer goods. Depending on the application, preservatives may be integrated directly into the product, such as in personal care and household items, or incorporated into the packaging, as is often the case with food products. However, many industries face challenges due to the high cost and limited effectiveness of current preservatives, along with growing regulatory restrictions on their use. For example, this is especially vital within the food industry where approximately $1 trillion is lost annually due to food spoilage ^1^, which contributes to nearly 10% of global greenhouse gas emissions ^2^. Reducing food waste and prolonging shelf-life are therefore urgent global priorities, with far-reaching economic, environmental, and social implications. These efforts align directly with the United Nations Sustainable Development Goals (UNSDGs), particularly Zero Hunger (UNSDG 2), Good Health and Well-being (UNSDG 3), and Responsible Consumption and Production (UNSDG 12) ^3^. Consequently, there is growing demand for innovative packaging solutions that enhance preservation through the integration of active functional materials.

A promising approach involves the use of antimicrobial and antioxidant additives, particularly colloidal silver (CS) particles, which are known for their potent antimicrobial and anti-inflammatory properties. CS is increasingly used in industries such as cosmetics ^4,5^, packaging, ^6–8^, pharmaceutical ^9–11^ and other industrial applications to extend product shelf-life and improve safety. In 2023, the global market for this industry was valued at $2.68 billion and is projected to reach around $4.45 billion by 2030, growing at a compound annual growth rate (CAGR) of 7.5% ^12^. Within this market, healthcare holds a significant share (34.9%), with applications in wound care, surgical implants, and medical devices, while food packaging accounts for about 15% of CS usage ^6,7,13^. The antimicrobial additives market for food packaging alone is currently valued at $12.73 billion, comprising 3.25% of the global food packaging industry, and is growing at a CAGR of 5.7%.

However, widespread application of CS in consumer products faces key challenges. Conventional CS particles often present toxicity concerns, high production costs, and declining antimicrobial efficacy over time. Preservation systems still commonly rely on biocides like Methylisothiazolinone (MIT), Triclosan, and Benzyl Alcohol, substances increasingly restricted due to their harmful health and environmental impacts ^14–16^. MIT, for instance, has been banned in leave-on cosmetics in the EU ^17^, and Triclosan is no longer allowed in food packaging ^18^. Additionally, antimicrobial resistance (AMR) and shifting consumer preferences are driving demand for safer, more sustainable solutions ^19^. CS stands out as one of the most promising technologies to meet these needs. Compared to traditional silver derivatives such as silver nitrate, CS particles show lower toxicity ^20^ and superior antimicrobial activity, likely due to their slower, sustained silver release ^21^. They are effective against more than 600 pathogens, including resistant strains ^22,23^. However, the method of synthesis significantly influences the properties and viability of CS in end-use applications.

Traditionally, CS has been synthesized using chemical reduction methods, where silver salts (typically silver nitrate) are reduced using agents such as sodium borohydride, ^24^ or hydrazine. These methods offer precise control over particle size and can produce relatively uniform, monodisperse particles. However, they often involve hazardous chemicals, generate toxic by-products, and require additional surface modifications to ensure biocompatibility and long-term stability. Furthermore, their reliance on toxic reagents and complex purification steps raises significant environmental and regulatory concerns ^25–27^. Physical methods, such as laser ablation and electrochemical synthesis ^28,29^, can produce high-purity particles without the need for chemical stabilizers, but they are energy-intensive, expensive, and difficult to scale. These limitations make conventional methods unsuitable for large-scale or consumer-safe production, especially in applications that demand eco-friendliness, regulatory compliance, and cost efficiency. Furthermore, these methods often produce particles smaller than 100 nm, limiting their use in consumer products under current EU and UK regulations, which restrict nanomaterials between 1–100 nm in food packaging and consumer applications ^30,31^.

Another significant hurdle is the scalability of CS production. Large-scale synthesis often leads to particle instability, aggregation, and loss of antimicrobial activity, particularly in industrial or physiological formulations ^32–34^. Maintaining consistent particle size and dispersion is difficult due to batch-to-batch variability and uncontrolled reaction kinetics ^35,36^. The lack of standardization in particle characterization also hinders the ability to assess efficacy and compare results across studies, impeding commercial implementation.

Green synthesis methods, by contrast, use natural reducing and stabilizing agents derived from plant extracts, microorganisms, or biopolymers. These techniques operate under mild reaction conditions and avoid toxic reagents, making them safer and more sustainable. The plant-based compounds, such as polyphenols, flavonoids, and tannins, not only reduce silver ions but also form organic coatings around the particles, enhancing stability and biocompatibility ^37,38^. Green-synthesized CS particles can be tailored to a wide size range (1–1000 nm) and generally exhibit spherical morphology ^39^. These materials show improved biocompatibility and added antioxidant properties, making them well-suited for consumer applications ^9,40^.

However, while green synthesis presents a promising, eco-friendly alternative to traditional methods, it is not without its challenges. Colloidal silver particles produced via simple green approaches, such as those using lemon juice (LJ) or green tea (GT) often suffer from limited colloidal stability, inconsistent size distributions, and ineffective properties. For example, CS_GT_ and CS_LJ_ typically show poor size control and limited stability, ^26,41^ resulting in inconsistent antimicrobial performance ^42^. Furthermore, the composition of these plant extracts can vary significantly with harvest time, geography, and extraction method, making reproducible scale-up difficult. Recent research has therefore shifted toward the use of standardized plant-extract blends and controlled, bioreactor-based synthesis protocols. These strategies enable tighter regulation over reaction parameters, such as pH, temperature, and extract concentration, resulting in more uniform particle size, improved reproducibility, and enhanced scalability. Such optimized green synthesis approaches mitigate many of the shortcomings associated with simpler extract-based methods and bring green-synthesized colloidal silver closer to commercial viability ^43,44^.

This study aims to evaluate the colloidal stability of a portfolio of green-synthesized particles and compare their performance to a standard commercial CS solution in various industrially relevant conditions. The patented green commercial method, utilising BX3 extract which is a blend of plants, combines green synthesis methods. This novel process could overcome current limitations associated with green methods. By assessing both antioxidant and antimicrobial activities, two critical biological functions, this research seeks to determine optimal formulation parameters and establish a standard approach to particle characterization. Such advancements are essential for enabling the commercial viability and large-scale implementation of CS as part of the global effort to reduce waste, ensure safety, and support sustainable development.

## Materials and methods

### Materials

Reference silver dispersion (CS_Ref_) of 100 nm particle size (730,777–25ML), silver nitrate (AgNO_3_) (85,229–50G), hydrochloric acid solution 1 M (HCl) (H9892–100ML), sodium hydroxide (NaOH) ( S5881–500G), 2,2-Diphenyl-1-picrylhydrazyl (DPPH) (D1932–5G), potassium chloride (KCl) (P9541–500G), di-sodium hydrogen phosphate dihydrate (71,643–250G), Resazurin sodium salt (199,303-5G), Potassium phosphate monobasic (P5655–100G), sodium chloride (NaCl) (S9625–500G), Tryptone (vegetable) (16,922–500G), Yeast Extract (Y1625–250G), and Meat Extract (70,164–500G) were purchased from Merck (UK). Ethyl alcohol completely denatured (CDA) (B2C Retail; 1L) was purchased from Amazon. Silver(I) acetate (F494137–100G) was purchased from Fluorochem (UK). Lemon Juice concentrate and green tea bags were purchased from local supermarkets.

### Green-CS preparation

**Fig. 1:**
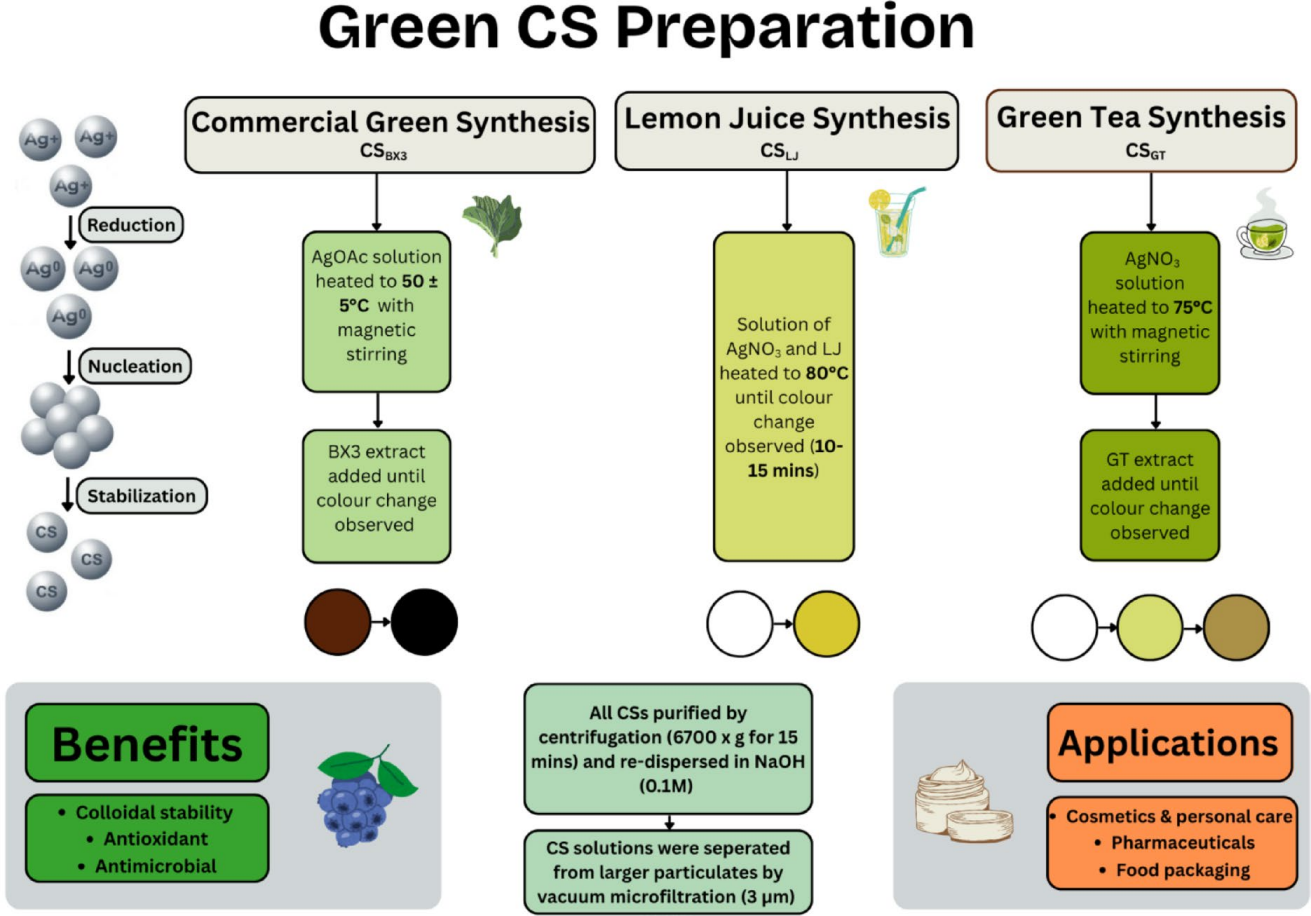
Schematic showing the synthesis steps for green CS method and a summary of the benefits of green synthesis methods and potential applications.

Each synthesis approach leveraged the diverse bioactive compounds present in natural extracts, which play a crucial role in both the reduction of silver ions (Ag⁺) to metallic silver (Ag⁰) and the stabilization of the resulting particles. The synthesis steps for each green CS method are summarized in Fig. 1.

#### Commercial green synthesis of CS (CS_BX3_)

Silver particles prepared from plant extracts were synthesised using the method outlined in the patented process with a plant extract mixture of oregano, kale, rosemary, artichoke and watercress in a proprietary formulation ^45^. Briefly, a solution of silver acetate was heated to 50 ± 5 °C under vigorous magnetic stirring. Metalchemy’s patented plant extract was subsequently added to achieve a final silver acetate concentration of 0.7 g/L. The reaction resulted in a rapid colour change of the solution from dark brown to black, signaling reaction completion. The resulting solution was then cooled in an ice bath. Particles were isolated by means of centrifugation (6700 × g for 15 min) and re-dispersed in sodium hydroxide (0.01 M). Silver particles in solution were separated from larger particulates by means of vacuum microfiltration (3 µm, Whatman grade 44).

#### Lemon juice synthesis of CS (CS_LJ_)

Silver particles were synthesised using an adapted method from Rajeshkumar et al. ^46^. A solution of silver nitrate (0.01 M) was added to commercially available lemon juice at a volume ratio of 4:1. The solution was subsequently heated to 80 °C and allowed to react for 10–15 min. The reaction was stopped when there was a colour change from colourless to yellow. Particles were isolated by means of centrifugation (6700 × g for 15 min) and re-dispersed in sodium hydroxide (0.01 M). Silver particles in solution were separated from larger particulates by means of vacuum microfiltration (3 µm, Whatman grade 44).

#### Green tea synthesis of CS (CS_GT_)

Silver particles were synthesised using an adapted method from Widatalla et al. ^47^. The green tea extract was first prepared by heating dried green tea in deionised (DI) water (at 10 g/L) to 60 °C. The extraction was carried out for 30 min under vigorous magnetic stirring. The solution was allowed to cool to room temperature and subsequently vacuum-filtered (3 µm Whatman filter papers) to separate the extract. To prepare the silver particles, a silver nitrate solution (10 mM) was heated to 75 °C under vigorous stirring. The green tea extract was added dropwise until a colour change from colourless to yellow-green to brown was observed. Particles were isolated by means of centrifugation (6700 × g for 15 min) and re-dispersed in sodium hydroxide (0.01 M). Silver particles in solution were separated from larger particulates by means of vacuum microfiltration (3 µm, Whatman grade 44).

### Colloidal silver particle characterisation

#### CS UV–Vis analysis

To analyse the CS solutions a Shimadzu UV-1900i spectrophotometer (Tokyo, Japan) was used to record UV–Vis spectra in the wavelength range of 300–600 nm at room temperature (25 °C). Variation in the peak absorbance was monitored under each test condition.

#### Scanning electron microscopy (SEM)

SEM was employed to analyze the surface characteristics and stability of each CS suspension. The samples were prepared by depositing a diluted CS suspension onto a silicon wafer using the drop-casting method. Following this, the samples were allowed to dry at room temperature for a duration of 24 h. A Zeiss Merlin FEG-SEM (Jena, Germany) Scanning Electron Microscope was used to obtain visuals across a range of magnifications spanning from 1000 × to 2000x. These images effectively depicted the morphology and aggregation behaviour of particles providing information on the stability of each CS suspension in various conditions.

#### Energy dispersive spectrometry (EDS)

EDS spectra were obtained using the Zeiss Merlin FEG-SEM equipped Oxford Instruments XMax EDS detector. The secondary electron detector was used for imaging. The voltages and currents were varied to switch between high-resolution imaging and analytics for EDS. The electron beam parameters were set, and EDS spectra were collected from multiple regions of the sample. EDX was utilized for the purpose of ascertaining the elemental composition of the particles. Furthermore, characteristic peaks present in the spectra allowed for the identification of any additional elements contained within the particles.

#### Particle size distribution and zeta potential

The size of silver particles and zeta potential (ZP) were determined using dynamic light scattering (DLS) with a Zetasizer Nano-ZS (Malvern Instruments, UK; model: ZEN3600, serial number: 100010754). Samples for size analysis were measured in disposable cuvettes ((Scientific Laboratory Supplies, UK; BR759005). Samples for ZP analysis were measured in folded capillary zeta cells (Malvern Panalytical, UK; DTS1070). Each sample was measured at 25 °C in triplicate.

#### Inductively coupled plasma mass spectrometry (ICP-MS)

Silver standards across a concentration range of 1–1000 ppb were prepared using a serial dilution method in triplicates. In addition, standards for each CS suspension (CS_BX3_, CS_LJ_, CS_GT_, and CS_Ref_) were prepared using a serial dilution method from the initial synthesised stock solution. Samples at day 0 and after 60 days storage at each temperature (4, 25 and 37 °C) were analysed to determine any change of silver concentration over time. To prepare these samples, 100 uL of the diluted suspensions used for the stability analysis, with a maximum absorbance of 1 circa (corresponding to ~ 20 ug/mL), were further diluted using DI water by a factor of 1000 to achieve solutions within a ppb concentration range. All samples were analysed using a Thermo Scientific iCAP-Q, run in Kinetic Energy Discrimination (KED) mode with a dwell time of 0.01 s.

#### Visual appearance

The visual appearance of CS suspensions was recorded by taking photographic images at day 0. Photographic images were taken using a Canon Rebel T7DLSR camera with 18–55 mm lens (Model Number: DS126741). Samples were placed in a light box DUCLAS photography light box.

### Colloidal silver stability assessment

Prior to the stability assessment, the synthesised silver particles were diluted using DI water, as required to achieve a maximum absorbance of ~ OD = 1 a.u circa (corresponding to ~ 20 µg/mL) in the range of 300–600 nm measured using a Shimadzu UV-1900i spectrophotometer. 3 mL of each suspension were measured in a 10 × 10 mm cuvette. BX3, LJ, GT, and Ref CS solutions were diluted using dilution factors of 5, 2, 1, and 1 respectively.

For all stability measurements, the surface plasmon resonance (SPR) band was measured using the same conditions and spectrophotometer. The hydrodynamic diameter (d_hyd_) and polydispersity index (PDI) for each diluted suspension was measured for a 1 mL volume in a 10 × 10 mm cuvette using a Malvern ZSU3100 Zetasizer. Each sample was measured in triplicate.

#### Long-term and temperature dependent stability of colloidal silver

The long-term stability of silver particles was investigated at 3 different temperatures; 5 mL of each suspension were stored at 4 °C, 25 °C (± 1 °C) and 37 °C (± 1 °C) monitoring possible changes in their hydrodynamic size (d_hyd_), polydispersity Index (PDI) and surface plasmon resonance (SPR) band in 7-day intervals over 60 days.

#### Phosphate-buffered saline stability

Silver particle stability was investigated by varying the ionic strength of the solutions. Their stability was investigated by UV–Vis analysis in PBS 1X (pH = 7.4) by injecting the diluted CS dispersions with 10% v/v PBS 10X (pH = 7.4). To prepare the 10X PBS solution, 100 mL of PBS was formulated by dissolving NaCl (1 g), KCl (0.025 g), Na_2_HPO_4_ (0.18 g) and K_2_HPO_4_ (0.0306 g) in 100 mL DI water. For stability testing, 2.7 mL of CS dispersion was mixed with 0.3 mL of PBS solution, resulting in a 10% dilution factor and the final concentration of 1X PBS in a total volume of 3 mL. Particle stability was monitored over time by recording the SPR bands in a wavelength range of 300–600 nm at regular time intervals of 0 min, 2 h, and 24 h. Particle size was determined using a Malvern ZSU3100 Zetasizer.

#### pH stability

The stability of silver particles was tested under acidic (pH 3), neutral (pH 7) and alkaline (pH 11) conditions. The pH of each CS suspension was adjusted by addition of 0.1 M, 0.05 M and 0.01 M solutions of HCl or NaOH. The pH of the suspension was measured by means of pH meter (± 0.01) (SevenDirect SD50 HA Kit, METTLER TOLEDO, Switzerland). The zeta potential (ZP) of each sample was measured using a Malvern ZSU3100 Zetasizer. Three measurements were carried out and an average with standard deviation was reported.

### Biological activity assessment

#### Antioxidant activity

The antioxidant activity of silver particles was determined using the DPPH (2,2-diphenyl-1-picrylhydrazyl) radical scavenging assay with minor adjustments ^48^. A 0.1 mM concentration stock solution of DPPH was prepared by dissolving 4 mg of DPPH in 100 mL of ethanol. Dilutions of CS were prepared in DI water at 2, 10, and 20 μg/mL. Each diluted CS suspension was mixed at a 1:1 volume ratio with the DPPH stock solution (yielding final CS concentrations of 1, 5, and 10 μg/mL) and incubated in the dark for 30 min. DPPH stock solution mixed with ethanol at a 1:1 ratio was used as a reference. The absorbance of the solutions was then measured at 517 nm using a spectrophotometer. The CS solutions contribute to the absorbance at 517 nm, therefore the absorbance of CS solutions, at each concentration, and mixed with EtOH in a 1:1 volume ratio was subtracted from the measured absorbance with DPPH. The percentage of DPPH radical scavenging activity was calculated using Eq. 1. The reaction was performed in triplicates and an average with standard deviation reported.

1 $$\text{\% DPPH inhibition}= \frac{{\text{A}}_{0} - {A}_{s}}{{A}_{0} }\times 100$$

where A_o_ = Control Absorbance, A_s_ = Sample Absorbance.

#### Antimicrobial activity

#### Minimum Inhibitory Concentration

The minimal inhibitory concentration (MIC) of silver particles was determined using an adapted protocol from Ahmad et al. ^49^. CS solutions were evaluated against the Gram-negative *bacterium Escherichia coli* (ATCC 25,922) and Gram-positive *bacterium Listeria innocua* (ATCC 33,090). Bacterial cultures were grown on LB-Nutrient agar plates for 24 h. The cultures were transferred to 1/50 nutrient broth suspension medium (SM) using a sterile inoculation loop. The resulting culture solution was adjusted to an optical density (O.D) at 600 nm of ~ 0.1 (corresponding to a final density of 1.2 X 10^7^ CFU/mL) using SM. The culture solution was further diluted, using SM, by × 100. The diluted bacterial suspension was added to CS in a 1:1 volume ratio to achieve final silver particle concentrations of 1.0, 2.5, 5.0, 7.5 and 10.0 µg/mL. The concentration of CS was capped at 10 µg/mL to simulate a biologically relevant dosage, ensuring that the findings could be extrapolated to potential therapeutic or environmental applications. Amoxicillin (10 µg/mL) and Ethanol (70%) were used as positive controls. Bacterium mixed with SM were used as negative controls. Samples were incubated at 36 °C for 24 h. The resazurin assay was performed to determine the percentage of dead/alive bacteria. A resazurin stock solution was prepared at 0.01 wt% in DI water. After 24 h incubation of the bacteria-CS samples at 36 °C, resazurin stock solution was added in a 1:10 volume ratio with each sample and incubated at 36 °C for 2 h. The O.D at 570 and 620 nm was measured for each sample and the % resazurin reduction determined using Eq. 2. Each experiment was performed in triplicate with an average and standard deviation reported.

2 $$\%\ Resazurin Reduction= \frac{{A}_{570 nm} - {A}_{620 nm}}{{A}_{570 nm}+ {A}_{620 nm}}\times 100$$

where A_570 nm_ = Absorbance at 570 nm (corresponding to Resorufin) and A_620 nm_ = Absorbance at 620 nm (corresponding to Resazurin).

### Statistical analysis

Quantitative data from the various methods were analysed by one-way analysis of variance (ANOVA) using Origin Software. For those measurements exhibiting significant difference in one way ANOVA, a Tukey’s post-hoc test was applied to determine which sample means differed significantly (p-value < 0.05).

## Results and discussion

### Green colloidal silver synthesis and characterisation

The size, morphology, and surface chemistry of colloidal silver (CS) particles are highly influenced by synthesis conditions such as pH, temperature, and the composition and concentration of reducing agents, which in turn affect their antimicrobial and antioxidant properties ^50,51^.

**Fig. 2:**
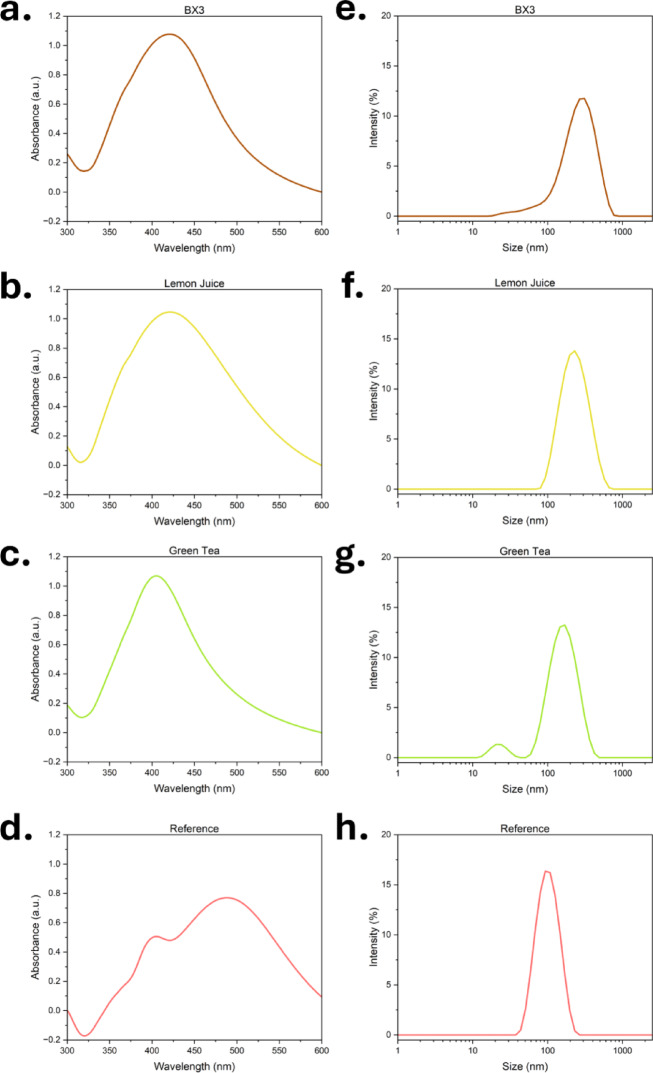
UV–Vis spectra of (**a**) CS_BX3_, (**b**) CS_LJ_, (**c**) CS_GT_, and (**d**) CS_Ref_ on day 0, recorded between 300 and 600 nm, showing characteristic SPR bands of silver particles. Particle Size Distribution (PSD) of (**e**) CS_BX3_, (**f**) CS_LJ_, (**g**) CS_GT_, and (**h**) CS_Ref_ measured on day 0.

Green synthesis methods using plant extracts successfully produced silver particles, confirmed by the presence of characteristic surface plasmon resonance (SPR) bands in UV–Vis spectra (Fig. 2a-d). The specific peak wavelength and absorbance values of the SPR bands for each CS suspension are summarized in Table 1. All green-synthesised CS showed single, well-defined SPR peaks, indicating monodispersity of silver particles in solution (Fig. 2a-c). The shift in SPR peaks indicate size and shape variations between synthesis methods. CS_BX3_ and CS_LJ_ had peaks near 420 nm, while CS_GT_ exhibited a blue-shifted SPR (~ 400 nm), suggesting smaller, potentially more reactive particles. CS_Ref_ displayed a broader dual-peak profile (~ 405 and ~ 480 nm) (Fig. 2d), consistent with larger particles ^52^. These spectral differences are reflective of variations in reducing/capping agents, causing differences in the reduction and nucleation profiles, affecting size and particle uniformity. For example, green tea contains catechins, polyphenolic compounds that strongly chelate silver ions, facilitating rapid reduction and stabilization of silver atoms. This leads to the early formation of numerous stable silver nuclei (seed particles), thereby shortening the nucleation phase and promoting the growth of smaller, more uniform particles ^53^.

**Table 1:** Summary of all key characteristics for each CS synthesis at day 0.

Characteristic	CS_BX3_	CS_LJ_	CS_GT_	CS_Ref_
Peak wavelength (nm)	421.0	422.5	405.5	404.5
Peak absorbance (a.u.)	1.077	1.046	1.071	0.506
d_hyd_ (nm)	250 ± 20	220 ± 10	180 ± 15	95 ± 10
pH	12.04	12.21	12.22	7.19
Zeta Potential (mV)	− 32.4 ± 0.3	− 12 ± 1	− 28.1 ± 0.8	− 35.4 ± 0.2
CS concentration by UV–Vis (μg/mL)	22 ± 2	22 ± 1	22 ± 2	11 ± 3
CS concentration by ICP-MS (μg/mL)	45 ± 3	43 ± 3	46 ± 2	35 ± 2
Appearance	Yellow-Amber	Yellow-Amber	Orange-Amber	Grey-White

DLS data showed CS_BX3_ and CS_LJ_ had hydrodynamic diameters (d_hyd_) between 220–250 nm (Table 1). Given their SEM-measured core size and monodisperse PSDs (Fig. 2e, f, g), the larger d_hyd_ is attributed to thick bioorganic coronas, derived from plant extracts, which offer steric hindrance and improved colloidal stability.^54^. These stabilizing layers also enhance biocompatibility, as supported by prior studies showing higher cell viability of CS_BX3_ in egg-shell membranes compared to CS_Ref_^9^. CS_Ref_ showed a narrow size distribution (~ 100 nm) (Fig. 2h), indicating better monodispersity due to controlled industrial synthesis. However, its commercial use may be restricted under nanomaterial regulations ^30,31^.

pH analysis (Table 1) revealed that CS_BX3_, CS_LJ_, and CS_GT_ were synthesized under highly alkaline conditions (pH > 12), which typically promote particle stability by enhancing the negative surface charge ^55^. Zeta potential (ZP) measurements supported this, with CS_BX3_ and CS_GT_, exhibiting ZP values around –30 mV (Table 1), indicative of good electrostatic stability. Hence the surface ligands bound to the particle surface originating from both these plant extracts (BX3 and GT) enable both steric and electrostatic stabilisation. In contrast, CS_LJ_ showed a significantly lower ZP of –12 ± 1 mV, suggesting weaker electrostatic repulsion. In this case, colloidal stability is likely maintained through steric hindrance provided by bulky, branched surface ligands derived from the lemon juice extract. CS_Ref_) which is citrate-stabilized and supplied at near-neutral pH (7.19), exhibited strong electrostatic stabilization with a ZP of –35.4 ± 0.2 mV. Here, the negative surface charge arises from the adsorbed citrate ions, which create sufficient repulsive forces to prevent aggregation.

**Fig. 3:**
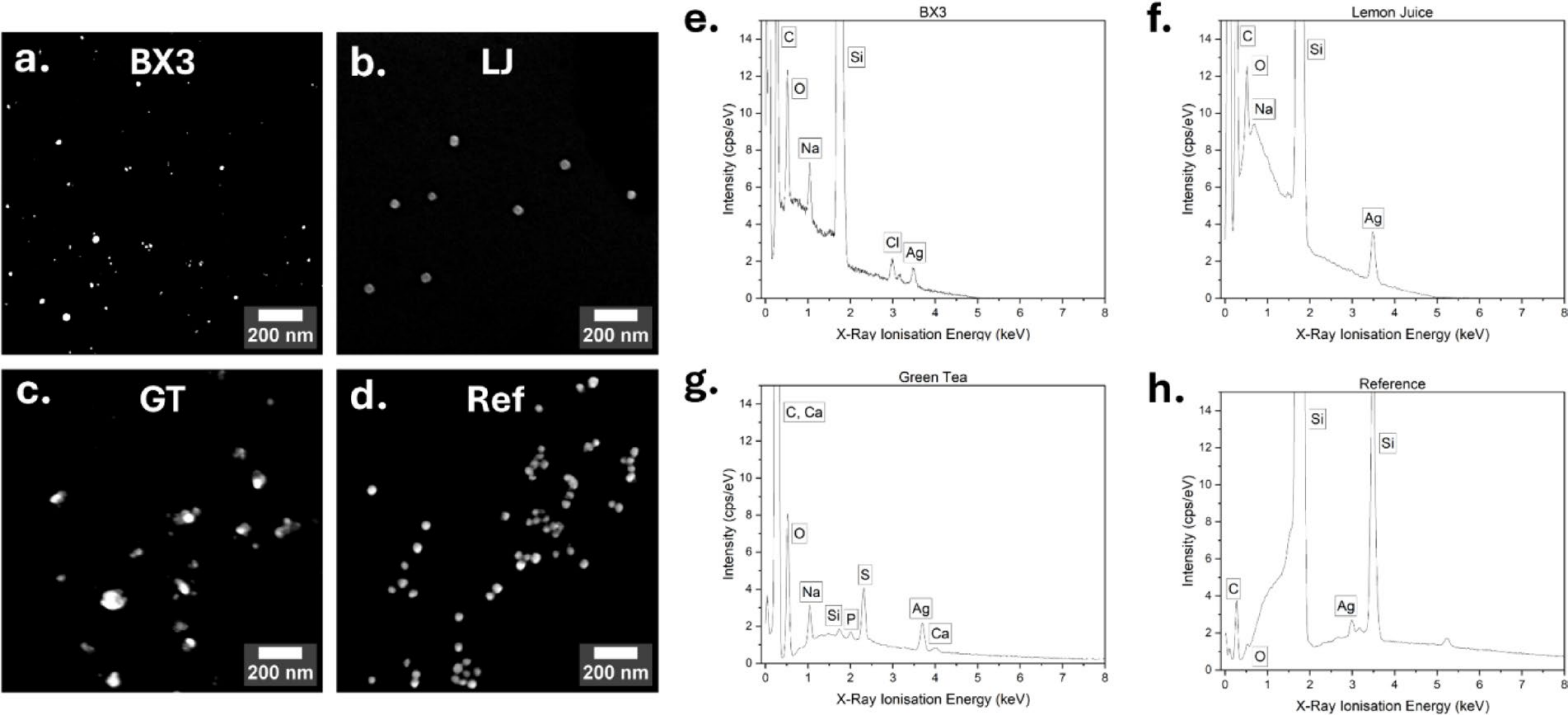
SEM images (**a**–**d**) of (**a**) CS_BX3_, (**b**) CS_LJ_, (**c**) CS_GT_, and (**d**) CS_Ref_ on day 0 captured using 50 K magnification. EDS spectra (**e**–**h**) of (**e**) CS_BX3_, (**f**) CS_LJ_, (**g**) CS_GT_, and (**h**) CS_Ref_ on day 0. Peaks are labelled with the characteristic elements. Samples were dispensed on Silica (Si) plates, explaining the high intensity peaks for Si.

Morphological analysis via SEM (Fig. 3a-d) confirmed the formation of predominantly spherical particles. CS_GT_ displayed significant aggregation, as seen in SEM (Fig. 3c) and DLS (Fig. 2g). We anticipate that this results from less effective or incomplete capping of bioorganic ligands on the particle surface. CS_Ref_ had the largest and most uniform particles, aligning with UV–Vis data.

Energy-Dispersive X-ray Spectroscopy (EDS) confirmed silver ion reduction in all samples, with Ag peaks around 3 keV (Fig. 3e-h) ^56^. CS_Ref_ exhibited two Ag peaks, possibly due to larger particle size or altered crystallinity ^57^. Organic elements (C, O) were also detected in green-synthesized CS solutions, confirming the presence of capping groups by plant-derived biomolecules such as polysaccharides or phenolics.

Overall, these findings highlight how plant-based green synthesis offers a versatile approach for tailoring CS particle properties. The choice of plant extract, synthesis parameters, and capping agents significantly impacts particle size, stability, and application potential. These physicochemical differences directly impact performance. CS_BX3_ and CS_LJ_, with uniform size and strong capping, are expected to offer controlled ion release, prolonged stability, and better biocompatibility, favorable traits for biomedical and personal care applications. Conversely, CS_GT_, while less stable, may be more catalytically active due to higher surface area and reactivity, making them suitable for environmental applications (e.g., pollutant degradation or antimicrobial coatings in water systems). CS_Ref_, while consistent in industrial synthesis, may face limitations due to regulatory restrictions and potential aggregation without adequate surface modification.

### Stability assessment

#### Long-term stability

**Fig. 4:**
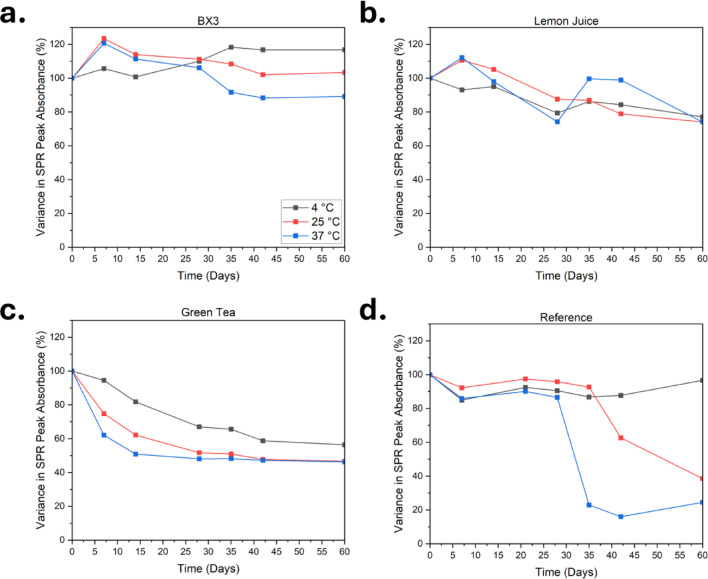
Absorbance variation of (**a**) CS_BX3_, (**b**) CS_LJ_, (**c**) CS_GT_, and (**d**) CS_Ref_ at storage temperatures of 4 °C, 25 °C, and 37 °C over a 60-day period. UV–Vis spectra were measured across a wavelength range of 300 to 600 nm at regular time intervals. The variation in the peak absorbance was determined in relation to the original peak absorbance at day 0.

The long-term stability of colloidal silver (CS) solutions is critical for functionality, bioavailability, and shelf life in practical applications. This study evaluated the stability of CS formulations by monitoring changes in their surface plasmon resonance (SPR) band (Fig. 4) and hydrodynamic diameter (d_hyd_) (Fig. S1) over 60 days under three storage temperatures (4 °C, 25 °C, and 37 °C).

CS_BX3_ demonstrated the highest stability, particularly at 4 °C, with only a 3.97% decrease in d_hyd_ and minimal SPR variation. At 25 °C and 37 °C, size reductions of 70–100 nm, with SPR absorbance showing progressive decline at 37 °C but remaining stable at 25 °C. Even at 37 °C, only a 10% loss in SPR absorbance was observed, indicating strong colloidal stability suitable for physiological applications. The observed size changes at elevated temperatures may be linked to core–shell restructuring or Ostwald ripening, a thermodynamically driven phenomenon where smaller particles undergo partial or full dissolution and redeposition onto larger particles ^58^. This process occurs due to differences in the solubility of smaller particles compared to larger particles ^59^. Initially, smaller particles shrink through dissolution of molecules at the surface of the particle which are energetically unstable. Once the solution is saturated with free molecules and the smaller particles have fully dissolved, the free molecules deposit onto the surface of larger particles. CS_LJ_ maintained consistent d_hyd_ across all temperatures but showed a 20% SPR decrease, suggesting effective steric stabilization and minor particle sedimentation. This stability is likely due to strong binding of bioactive compounds present in lemon juice ^60^. In contrast, CS_GT_ exhibited significant instability, with large size fluctuations and broadening SPR peaks across all temperatures, indicating rapid aggregation, especially at higher temperatures. CS_Ref_ remained stable at 4 °C and 25 °C but aggregated after 30 days at 37 °C, limiting its use in physiological environments. Overall, CS_BX3_ showed the best long-term stability, followed by CS_LJ_ and CS_Ref,_ while CS_GT_ was the least stable under all tested conditions.

#### Stability in phosphate-buffered saline

UV–Vis (Fig. S2) and DLS (Fig. S3) measurements were used to assess the stability of CS formulations over 24 h in PBS (1X), with results summarized in Table S1. All formulations showed an immediate drop in SPR peak absorbance upon PBS exposure (Fig. S2), indicating salt-induced charge screening that weakened electrostatic repulsion and promoted aggregation. CS_BX3_ showed the least reduction (14.2%), suggesting stronger resistance to ionic interactions compared to other formulations (25–47% reduction).

After 24 h, CS_LJ_ exhibited the greatest stability with only a 24.7% decrease in SPR intensity. This observation further confirms that particle stability in this case results predominantly from steric interactions. CS_BX3_ and CS_Ref_ showed moderate stability (35–44% reduction), while CS_GT_ were the least stable, with an 81.2% drop in SPR intensity and peak broadening.

DLS results supported these findings. CS_LJ_ had minimal size change (+ 29 ± 2 nm), and CS_Ref_ showed no significant size variation, indicating limited aggregation despite some sedimentation. CS_BX3_ displayed the largest increase in size (+ 154 ± 18 nm), suggesting substantial aggregation. CS_GT_ decreased in size (− 40 ± 9 nm) but developed a secondary peak, indicating aggregation into distinct clusters.

SEM images (Fig. S4) confirmed these trends: CS_LJ_ remained well-dispersed, CS_BX3_ and CS_Ref_ showed minor clustering, and CS_GT_ exhibited substantial agglomeration, reinforcing its poor colloidal stability in physiological conditions.

#### pH stability

**Fig. 5:**
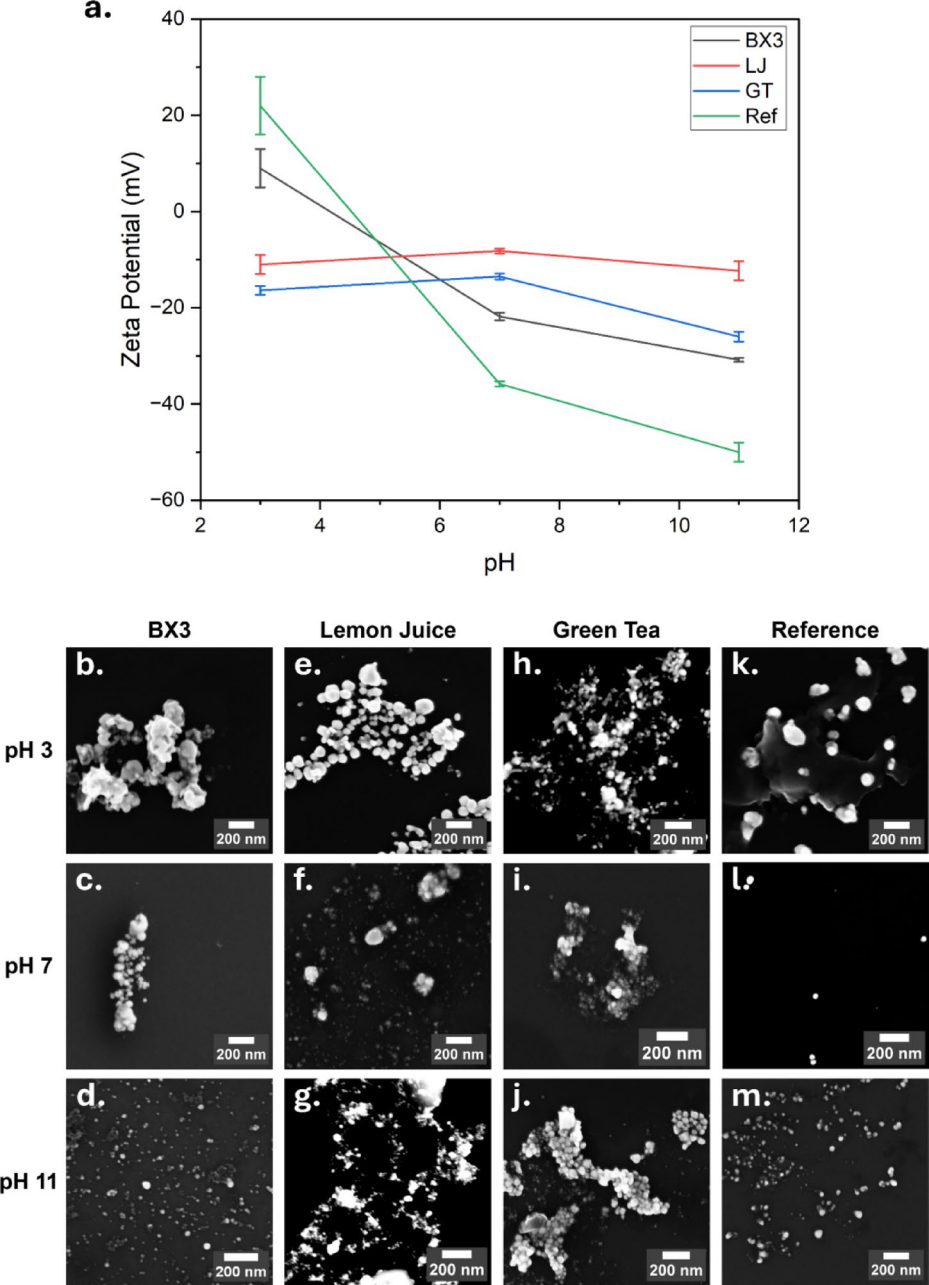
(**a**) Influence of pH on ZP at pH 3, 7 and 11 for CS_BX3_, CS_LJ_, CS_GT_, and CS_Ref_. **b**–**m** SEM Images of CS_BX3_, CS_LJ_, CS_GT_, and CS_Ref_ at pH 3 (**b**, **e**, **h**, **k**), pH 7 (**c**, **f**, **i**, **l**) and pH 11 (**d**, **g**, **j**, **m**) captured using 50 K magnification. CS solutions were adjusted to each pH using NaOH and HCl solutions. ZP measurements were carried out 2 h after pH adjustment. Measurements were performed in triplicate and an average and standard deviation determined. Statistical analysis was performed using ANOVA 1-way analysis and Tukey test. At each pH, ZP values for each CS suspension were determined to be significantly different, except for LJ and GT at pH 3.

Zeta potential (ZP) analysis revealed distinct stability profiles for different CS formulations under varying pH conditions (Fig. 5a, Table S2). CS_Ref_ and CS_BX3_ follow similar trends in their zeta potential vs pH stability curves. CS_BX3_ demonstrated the highest stability at pH 11, with a ZP of –30.8 ± 0.4 mV, only a 27.7% reduction in SPR absorbance, and a minor size change (+ 10 ± 3 nm) compared to its synthesis pH of 12.04. At pH 7, ZP dropped to –21.8 ± 0.8 mV, leading to moderate aggregation (64.3% SPR reduction, + 30 ± 18 nm size), consistent with destabilization near the isoelectric point ^61^. Despite this, a portion of ~ 100 nm particles remained suspended, suggesting moderate stability. CS_GT_ showed moderate surface charge at pH 11 (–26 ± 1 mV) but still experienced a 78.5% loss in SPR absorbance, indicating aggregation despite minor size changes. At pH 7 and 3, ZP dropped below –20 mV, with SPR reductions of 95% and size increases of + 48 ± 8 nm and + 203 ± 21 nm, respectively, suggesting instability due to surface protonation, weakening electrostatic repulsion ^62^. CS_LJ_ had consistently low ZP (~ –10 mV) across all pH values, pointing to weak electrostatic stabilization and a high tendency for aggregation. In contrast, CS_Ref_ remained stable at neutral and alkaline pH (ZP < –35 mV), with < 40% SPR reduction and only 10–15 nm size increase. At pH 3, ZP shifted to + 20 mV, but CS_Ref_ still outperformed green-synthesized particles in stability under acidic pH. The tendency of silver particles to aggregate at acidic pH has been well-documented, as lower pH conditions weaken the negative charge on the particle surface, allowing Van der Waals forces to dominate ^63^.

SEM images (Fig. 5b–m) confirmed these findings. All formulations aggregated at pH 3, but CS_BX3_ and CSs_Ref_ remained more dispersed at pH 7 and 11. The acidic instability highlights the need for stabilizing ligands like PEG or chitosan^64,65^.

These findings have application-specific implications: CS_Ref_ and CS_BX3_, with moderate to strong neutral pH stability, are better suited for biomedical and personal care products, whereas less stable CS_GT_ and CS_LJ_ may be limited in antimicrobial applications such as food packaging or household products.

### Biological activity

#### Antioxidant activity

**Fig. 6:**
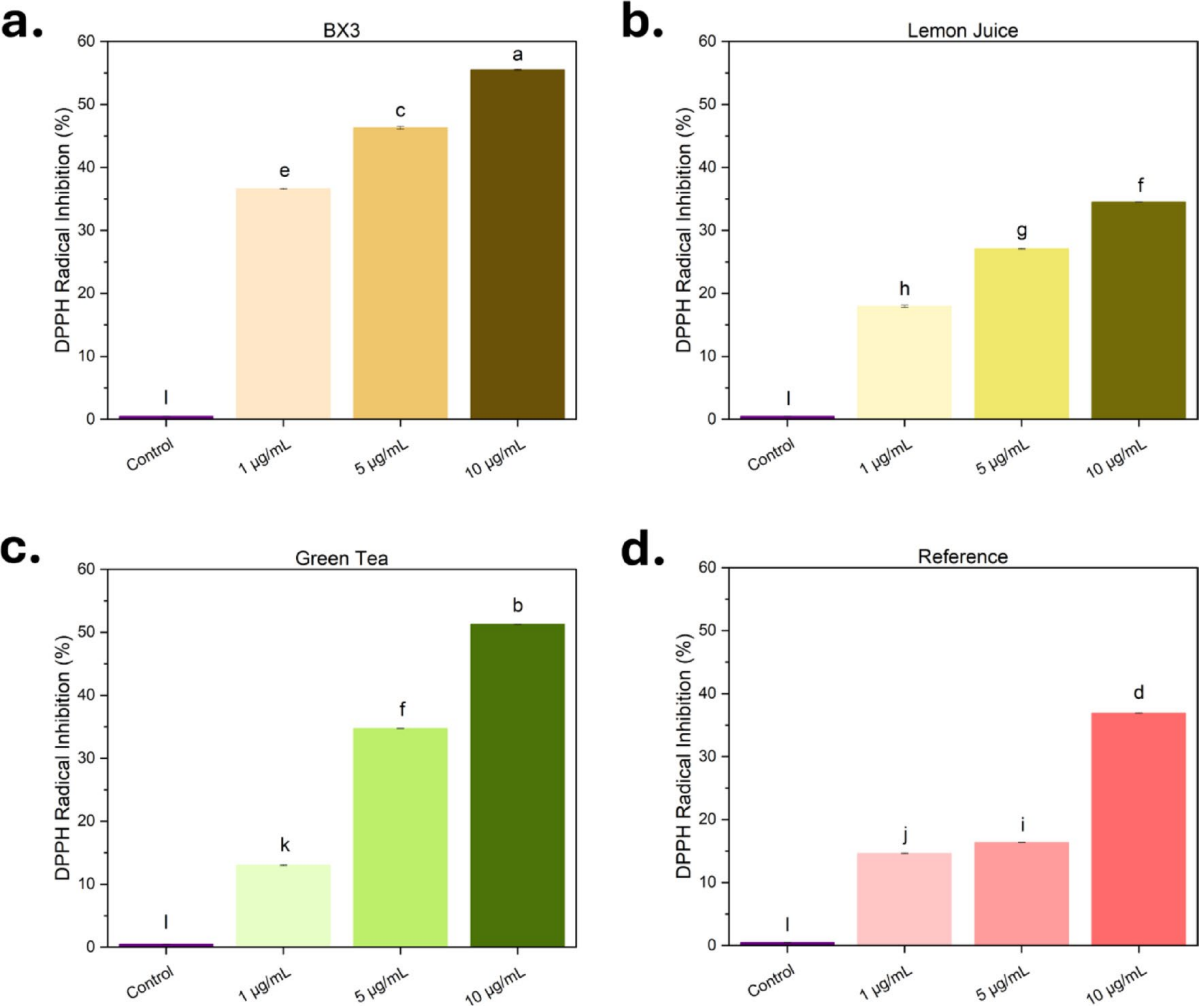
% Inhibition of DPPH free radical scavenging activity of (**a**) CS_BX3_, (**b**) CS_LJ_, (**c**) CS_GT_, and (**d**) CS_Ref_ at different concentrations (1–10 µg/mL). CS solutions were mixed in a 1:1 ratio with the DPPH solution. Optical density at 517 nm was measured after 30 min incubation. Radical inhibition was determined using Eq. 1. Statistical analysis was performed using ANOVA 1-way analysis and Tukey test. The radical scavenging activity of CS suspensions was determined to be significantly different, except for LJ at 10 µg/mL and GT at 5 µg/mL, which were statistically similar.

The antioxidant activity of CS suspensions was evaluated across concentrations of 1–10 µg/mL (Fig. 6). All formulations demonstrated measurable antioxidant properties, confirming their ability to neutralize oxidative stress, critical in biological and environmental applications.

CS_BX3_ showed the highest antioxidant activity across all concentrations, approximately 20% more effective than CS_LJ_ and CS_Ref_. At 10 µg/mL, CS_GT_ achieved ~ 50% radical inhibition, close to CS_BX3_ (~ 56%). This is likely due to a high concentration of catechins in the BX3 extract and green tea, known for their antioxidant potential ^53^. The superior activity of CS_BX3_ may result from a higher content of phytochemicals or flavonoids in the BX3 extract, enhancing the electron-donating capacity of the nanoparticles ^66^. This highlights that combining multiple plant materials in the extract can enhance the functional properties of the resulting particles, particularly valuable for applications in personal care and food packaging, where antioxidant activity plays a critical role in product performance. Additionally, the smaller core particle size increases the surface area, promoting better interaction with reactive oxygen species (ROS) ^67^.

CS_BX3_ and CS_GT_ demonstrated the strongest antioxidant performance, with IC50 values of 8.9 and 9.5 µg/mL, respectively. CS_LJ_ and CS_Ref_ were less active, with extrapolated IC50 values of 18.2 and 16.1 µg/mL. However, CS_LJ_ showed improved antioxidant activity compared to previous studies (IC50 of ~ 42 µg/mL ^60^), as well as other green synthesis methods (IC50 of ~ 30 µg/mL ^68^) likely due to enhanced morphology and stability from the optimized synthesis methods. Overall, these results highlight the key role of plant-derived compounds and secondary metabolites in boosting the antioxidant capabilities of green-synthesized silver particles ^69–71^.

#### Minimum inhibitory concentration

The antimicrobial activity of CS suspensions was evaluated against *E.coli* and *Listeria* using the resazurin assay (Fig. S5). Positive controls, Amoxicillin (10 µg/mL) and 70% Ethanol, showed complete bacterial eradication. All CS formulations exhibited antimicrobial effects, but only CS_BX3_ (≥ 10 µg/mL) and CS_GT_ (≥ 5 µg/mL) reached minimum inhibitory concentrations (MICs) after 24 h, demonstrating potent bactericidal activity comparable to the controls. CS_LJ_ and CS_Ref_ showed weaker effects, with ~ 30–40% bacterial survival.

The strong antimicrobial properties of CS_BX3_ and CS_GT_ likely result from the synergistic action of silver ions and plant-derived bioactive compounds, such as flavonoids, phenolics, and carotenoids ^72^. These natural compounds enhance silver’s biocidal effects by disrupting bacterial membranes and metabolism ^60^.

These findings highlight the effectiveness of green-synthesized silver particles, particularly from GT and BX3, and support their potential as dual-action antimicrobial agents for biomedical, packaging, personal-care and general consumer applications.

### Overall discussion

The findings of this study have direct relevance to real-world industrial, biomedical, and environmental applications. Each of the green-synthesized colloidal silver formulations, CS_BX3_, CS_LJ_, and CS_GT_, exhibited distinct physicochemical and biological profiles that align with specific commercial use cases. CS_BX3_ demonstrated exceptional long-term colloidal stability and strong antioxidant and antimicrobial performance, suggesting its suitability for high-value applications such as food packaging, personal care products, and topical antimicrobial treatments. Its ability to maintain structural integrity under a variety of stress conditions, such as temperature fluctuation, pH variation, and prolonged storage, indicates promise for use in both ambient and refrigerated supply chains, where product preservation and shelf-life are critical concerns. CS_LJ_, while displaying slightly lower bioactivity, maintained outstanding stability in physiological media such as phosphate-buffered saline (PBS), supporting its potential for biomedical applications including wound care materials, gels, and antiseptic formulations. Although CS_GT_ was the least stable under stress conditions, its pronounced antimicrobial activity and relatively high antioxidant capacity could make it a suitable candidate for short-term or disposable applications, such as antimicrobial coatings, water purification systems, or surface sprays, where immediate bioactivity is prioritized over long-term dispersion stability.

Compared to previous work in the field, this study introduced several important advancements. First, we utilized a standardized and patented plant extract blend (CS_BX3_) rather than single-source extracts. This enabled better reproducibility, more consistent particle properties, and improved scalability, overcoming a major limitation in many green colloidal silver particle syntheses. Second, we applied a comprehensive battery of stress tests to simulate real-world storage and use environments. This included evaluating particle stability under temperature variations, extended storage times, a wide pH range, and physiological salt concentrations. These conditions closely mirror the challenges encountered in consumer and medical product formulations, providing valuable insight into the behavior of these materials beyond idealized laboratory settings.

Furthermore, the biological activity of the synthesized particles was quantitatively assessed using both antioxidant and antimicrobial assays, producing robust and directly comparable data across samples. Notably, CS_LJ_ demonstrated improved antioxidant efficacy compared to prior literature (IC50 ~ 18.2 µg/mL versus previously reported values around ~ 42 µg/mL), a result attributed to improved control of particle morphology and surface capping during synthesis. The study also made deliberate efforts to design particles with hydrodynamic diameters greater than 100 nm, a key consideration given current EU regulatory restriction on the use of nanomaterials smaller than 100 nm in food and consumer products. This adjustment ensures greater regulatory compatibility and de-risks future product development efforts.

Taken together, these results support a clear conclusion: while simple, low-cost natural methods using single plant extracts such as lemon juice (LJ) and green tea (GT) can yield functional silver particles, they consistently underperform across multiple metrics compared to the CS_BX3_ formulation. Specifically, LJ and GT methods resulted in lower overall antioxidant and antimicrobial activity, reduced long-term stability, greater susceptibility to aggregation under stress, and inconsistent synthesis control. These limitations pose significant challenges for commercial scalability and quality assurance. The superior performance of CS_BX3_ across biological and physicochemical evaluations highlights the necessity of more refined and standardized synthesis protocols, especially when targeting high-value applications that require regulatory compliance, batch-to-batch consistency, and extended shelf-life.

The translational implications of these results are substantial. By directly linking synthesis parameters to stability and biological performance, this work enables targeted application of specific formulations based on functional needs. CS_BX3_ stands out as a versatile, high-performance candidate for commercial use, CS_LJ_ may be better suited for clinical or biocompatible applications, and CS_GT_ offers potential in time-limited or environmentally focused technologies. Importantly, these findings also contribute toward defining standard testing frameworks for green materials, which are currently lacking in the field.

To advance these materials toward commercialization, future research should focus on cytotoxicity profiling, in vivo compatibility studies, and regulatory compliance evaluations. Integration trials in packaging films, wound dressings, and other product matrices will be necessary to confirm material performance in applied settings. Additionally, techno-economic analysis (TEA) and life cycle assessment (LCA) should be performed to validate the environmental and economic sustainability of these green synthesis pathways. Collectively, these next steps will support broader market adoption of eco-friendly silver nanoparticle technologies capable of addressing global challenges in waste reduction, public health, and sustainable manufacturing.

## Conclusions

In conclusion, CS_BX3_ demonstrated the most balanced performance across all formulations, offering superior long-term stability, high antioxidant and antimicrobial activity, and promising applicability in food packaging, personal care, and biomedical products as a one-stop solution for product protection. While CS_LJ_ showed excellent biological stability, their lower bioactivity and pH sensitivity may limit broader use. CS_GT_ exhibited strong antimicrobial effects but suffered from poor stability and synthesis control, whereas CS_Ref_, despite good pH and PBS stability, lacked both long-term stability and biological efficacy. Among all, BX3 emerged as the most commercially viable candidate.

Future work should focus on regulatory approvals, cytotoxicity assessments, and scaling up production. Improving stability in lower pH environments would broaden silver particle solutions synthesised using BX3 application scope. Additionally, techno-economic analyses and Life Cycle Assessments (LCAs) will be essential to validate environmental and commercial sustainability, helping to accelerate market adoption of green-synthesized silver particles.

## Supplementary Information

Below is the link to the electronic supplementary material.

Supplementary Material 1

## Data Availability

Data is provided within the manuscript or supplementary information files.

## Abbreviations

CS: Colloidal Silver
LJ: Lemon Juice
GT: Green Tea
Ref: Reference
DLS: Dynamic Light Scattering
d_hyd_: Hydrodynamic Diameter
PBS: Phosphate Buffered Saline
PCPs: Personal Care Products
ZP: Zeta Potential
SPR: Surface Plasmon Resonance
PSD: Particle Size Distribution
UNSDG: United Nations Sustainable Development Goal
UV–Vis: Ultraviolet–Visible Spectroscopy
SEM: Scanning Electron Microscopy
EDS: Energy Dispersive X-ray Spectroscopy
