# Supplementary material for: A comparative evaluation of green colloidal silver variants for enhanced stability and bioactivity
Source: Sci Rep. 2025 Dec 5;16:747. doi: 10.1038/s41598-025-30332-7 (PMC12779990; doi:10.1038/s41598-025-30332-7)
Supplement: Supplementary file 1 — Supplementary Material 1 [file 41598_2025_30332_MOESM1_ESM.docx]

# Supplementary Information

# A Comparative Evaluation of Green Colloidal Silver Variants for Enhanced Stability & Bioactivity

# Federico Trotta^1,*^, Danielle Winning^1^, Sophie Sadiatoonasa^1^, Seyedeh Fatemeh Mirpoor ^2^, Stella Lignou^2^, Sameer Khalil Ghawi^2^ and Dimitris Charalampopoulos^2^

#### ^1^Metalchemy Limited, 71-75 Shelton Street, London WC2H 9JQ, UK

#### ^2^Department of Food and Nutritional Sciences, University of Reading, P.O. Box 226, Whiteknights, Reading RG6 6AP, UK

#### ^*^Author to whom correspondence should be addressed (ft@metalchemy.tech).


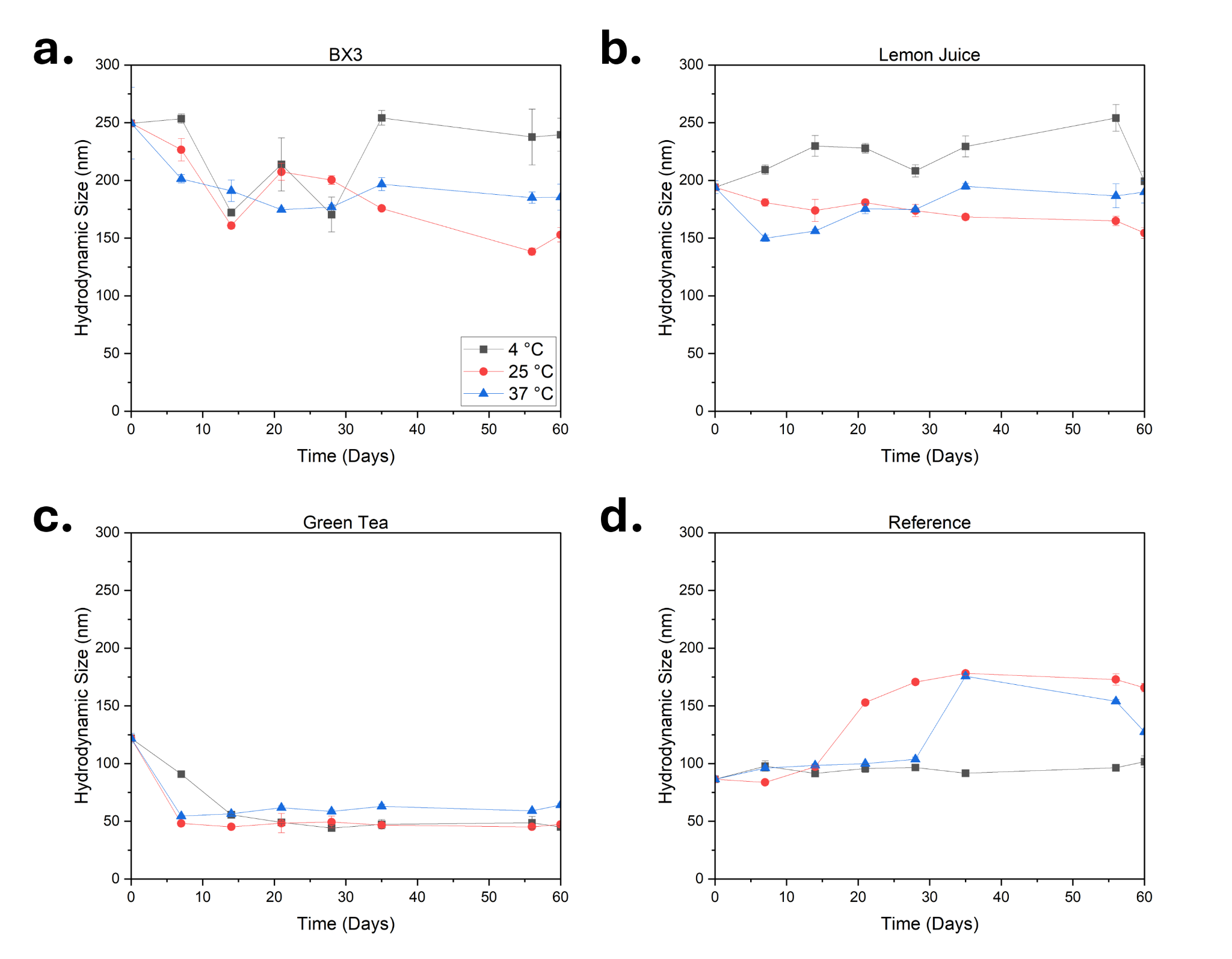


Figure S1: Time-dependent hydrodynamic size of a. CS_BX3_, b. CS_LJ_, c. CS_GT_, and d. CS_Ref_ at storage temperatures of 4 °C, 25 °C, and 37 °C measured at regular intervals over a 60-day period. Measurements were carried out three times and an average with standard deviation determined.


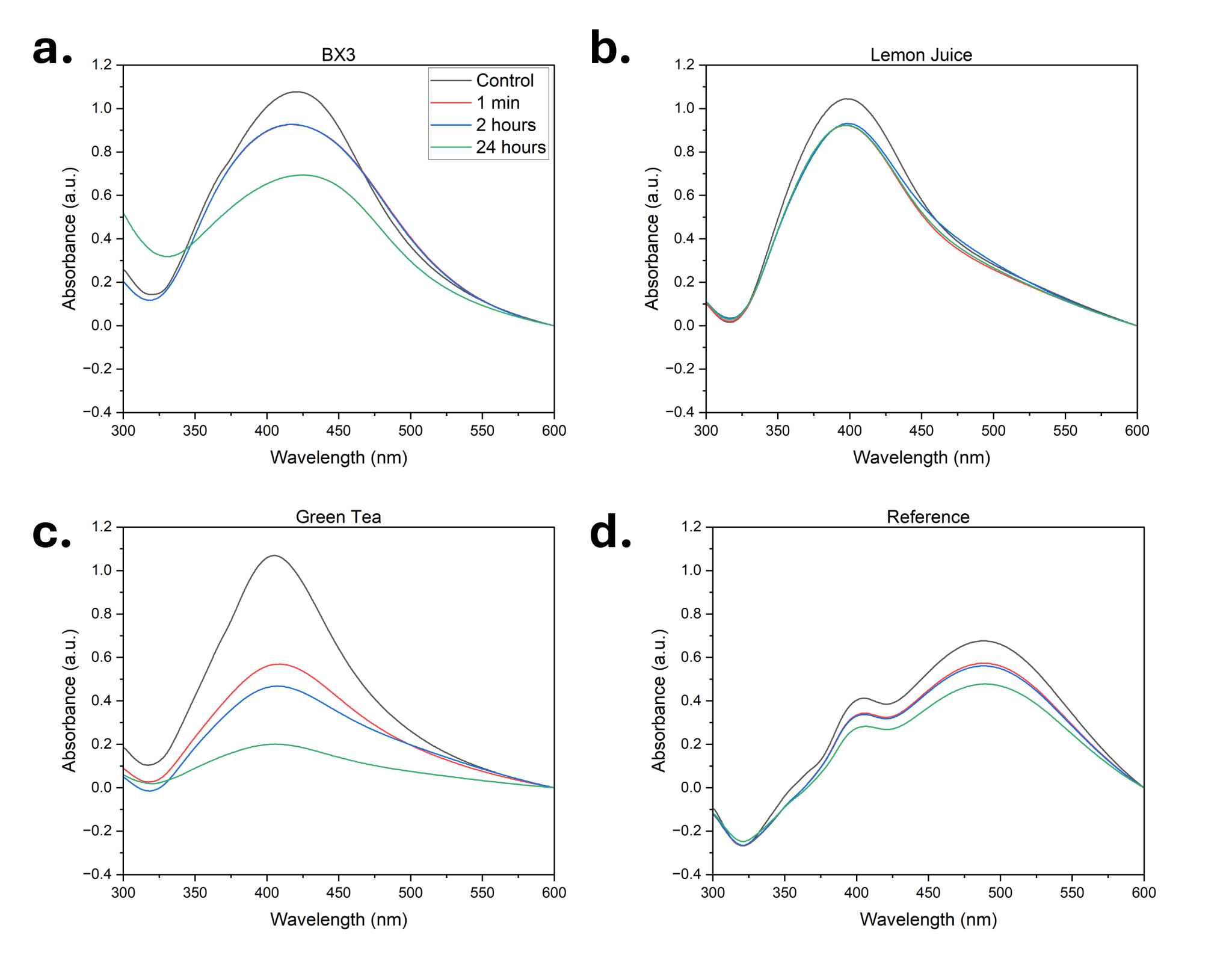
Figure S2: UV-Vis Spectra of a. CS_BX3_, b. CS_LJ_, c. CS_GT_, and d. CS_Ref_ in 10X PBS after 1 minute, 2 hours, and 24 hours.


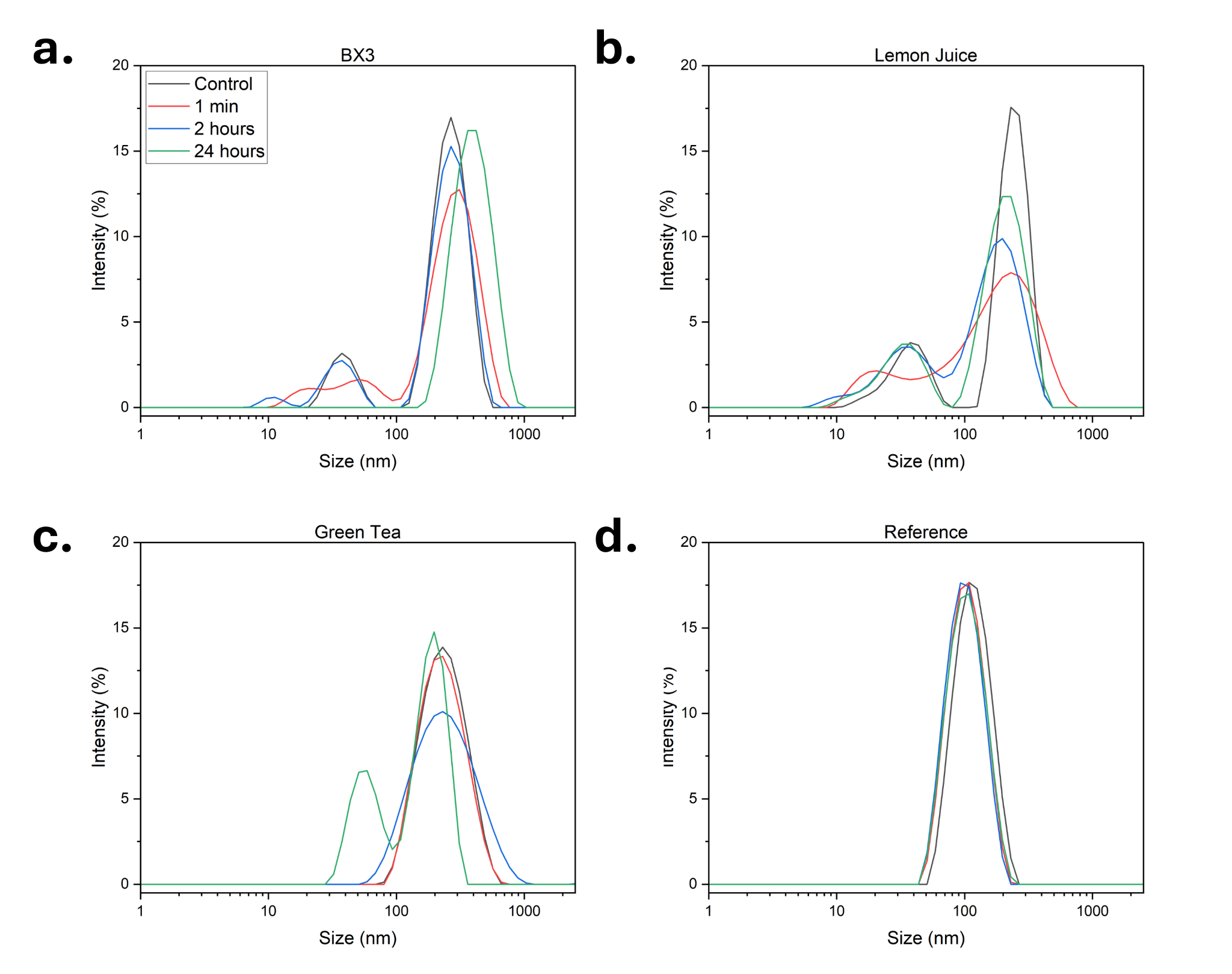
Figure S3: Particle size distribution (PSD) of a. CS_BX3_, b. CS_LJ_, c. CS_GT_, and d. CS_Ref_ in 1X PBS after 0 minutes (control), 1 minute, 2 hours, and 24 hours.

Table S1: Summary of changes in SPR peak absorbance and d_hyd_ of CS suspensions after incubation with PBS (1X) for 24 hours.

| **CS Suspension** | **Variation in SPR peak absorbance (%)** | **Variation in d_hyd_ (nm)** |
| --- | --- | --- |
| BX3 | -35.7 | +154±18 |
| LJ | -24.7 | +29±2 |
| GT | -81.2 | -40±9 |
| Ref | -44.1 | -2±1 |


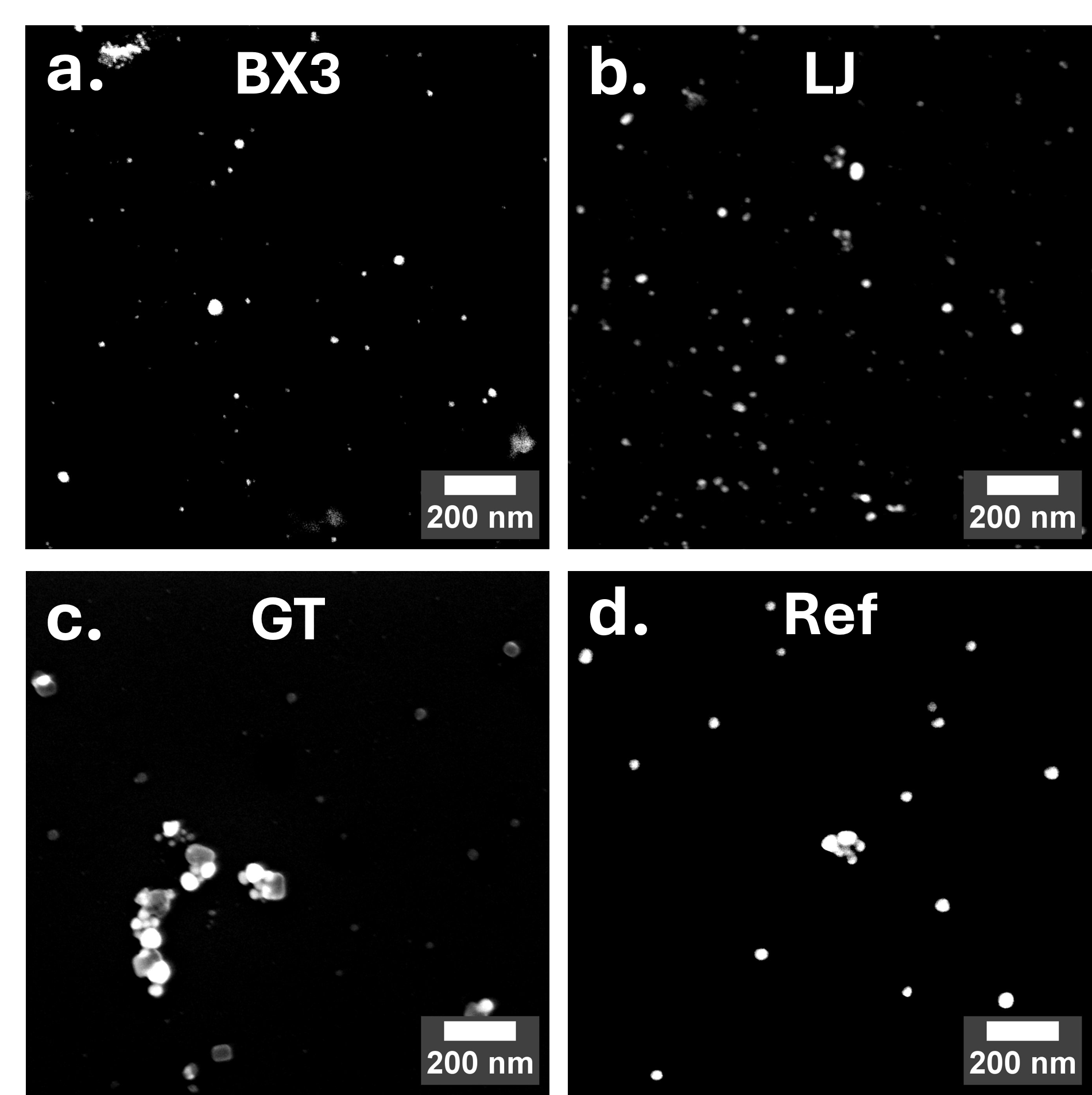


Figure S4: SEM images of a. CS_BX3_, b. CS_LJ_, c. CS_GT_, d. CS_Ref_, in 1X PBS for 24 hours captured using 50K magnification.

Table S2: Summary of changes in SPR peak absorbance and d_hyd_ of CS suspensions after pH adjustment to pH 3.0, 7.0 and 11.0 from initial pH (>pH 12 for all green synthesised CS) and pH 7.2 for Reference).

| **CS Suspension** | **pH** | **Variation in Peak Absorbance (%)** | **Variation in d_hyd_ (nm)** |
| --- | --- | --- | --- |
| BX3 | 3 | -70.3 | +109±18 |
|  | 7 | -64.3 | +30±18 |
|  | 11 | -27.7 | -10±3 |
| LJ | 3 | -96.9 | +480±37 |
|  | 7 | -73.6 | -57±17 |
|  | 11 | -50.3 | -40±8 |
| GT | 3 | -95.5 | +203±21 |
|  | 7 | -94.9 | +46±8 |
|  | 11 | -78.5 | -23+9 |
| Ref | 3 | -42.9 | -16±4 |
|  | 7 | -24.3 | +10±2 |
|  | 11 | -37.9 | +15±2 |


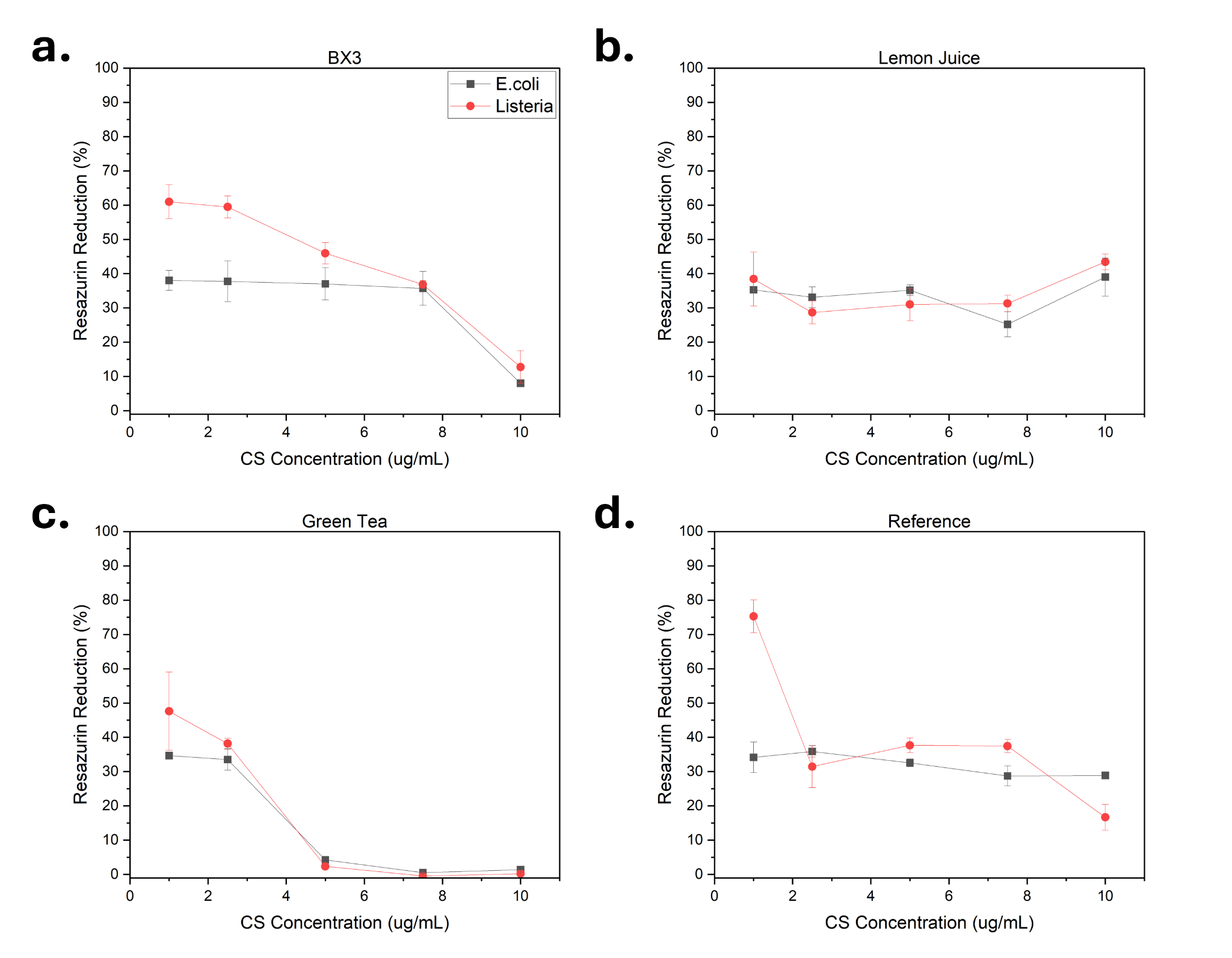


Figure S5: % Resazurin reduction of *E. coli* and *Listeria* incubated with a. CS_BX3_, b. CS_LJ_, c. CS_GT_, and d. CS_Ref_ at different concentrations (1-10 µg/mL). CS solutions were mixed in a 1:1 ratio with the *E. coli* or *Listeria* suspension. After 24 hours incubation at 36 °C, resazurin solution (0.01 wt%) was added to the mixed CS-bacteria suspensions in a 1:10 ratio. Samples were kept in the dark at 36 °C for 2 hours. Optical density at 570 and 620 nm was measured and the resazurin reduction calculated using Equation 2.

##### 
